# Burchellin: study of bioactivity against *Aedes aegypti*

**DOI:** 10.1186/1756-3305-7-172

**Published:** 2014-04-08

**Authors:** Juliana Oliveira Abreu Narciso, Renata Oliveira de Araújo Soares, Jacenir Reis dos Santos Mallet, Anthony Érico Guimarães, Maria Célia de Oliveira Chaves, José Maria Barbosa-Filho, Marise Maleck

**Affiliations:** 1 Laboratório de Diptera, Instituto Oswaldo Cruz, FIOCRUZ, Av. Brazil 4365, Rio de Janeiro 21045-900, Brazil; 2 Laboratório de Biologia Molecular e Doenças Endêmicas, Instituto Oswaldo Cruz, FIOCRUZ, Av. Brazil 4365, Rio de Janeiro 21040-360, Brazil; 3 Laboratório de Transmissores de Leishmanioses, Setor de Entomologia Médica e Forense, Instituto Oswaldo Cruz, Av. Brasil 4365, 21040-360 Rio de Janeiro, Brazil; 4 Laboratório de Tecnologia Farmacêutica, Universidade Federal da Paraíba, C. P. 5009, 58051-970 João Pessoa, PB, Brazil; 5 Laboratório de Insetos Vetores, Ciências da Saúde and Mestrado Profissional em Ciências Ambientais, Universidade Severino Sombra, Av. Expedicionário Oswaldo de Almeida Ramos, 280, 27700-000 Vassouras, RJ, Brazil

**Keywords:** Dengue, *Aedes aegypti* (L.), Neolignan burchellin, *Ocotea cymbarum* (Lauraceae), Larvicidal activity

## Abstract

**Background:**

The dengue mosquito *Aedes aegypti* Linnaeus, 1762 is a widespread insect pest of serious medical importance. Since no effective vaccine is available for treating dengue, the eradication or control of the main mosquito vector is regarded as essential. Since conventional insecticides have limited success, plants may be an alternative source of larvicidal agents, since they contain a rich source of bioactive chemicals. The aim of this study was to evaluate the larvicidal activity of the neolignan burchellin isolated from *Ocotea cymbarum* (Lauraceae), a plant from the Amazon region, against third instar larvae of *A. aegypti.*

**Methods:**

Burchellin obtained from *O. cymbarum* was analyzed. The inhibitory activity against *A. aegypti* eggs and larvae and histological changes in the digestive system of treated L3 larvae were evaluated. In addition, nitric oxide synthase activity and nitric oxide levels were determined, and cytotoxicity bioassays performed.

**Results:**

The data showed that burchellin interfered with the development cycle of the mosquito, where its strongest toxic effect was 100% mortality in larvae (L3) at concentrations ≥ 30 ppm. This compound did not show target cell toxicity in peritoneal macrophages from BALB/c mice, and proved to have molecular stability when dissolved in water. The L3 and L4 larvae treated with the compound showed cellular destruction and disorganization, cell spacing, and vacuolization of epithelial cells in small regions of the midgut.

**Conclusion:**

The neolignan burchellin proved to be a strong candidate for a natural, safe and stable phytolarvicidal to be used in population control of *A. aegypti*.

## Background

*Aedes* (Stegomyia) *aegypti* Linnaeus, 1762, whose main medical importance is due to its spread in urban areas and vector capacity for dengue virus [1], is responsible for frequent epidemics caused by the migration of the four serotypes within the Americas [2]. Females of the classic dengue vector, *A. aegypti* distribute their eggs among several oviposition sites and have a great capacity for adaptation to adverse conditions [3,4], making the control of this vector very difficult. The application of insecticides is undermined by its diurnal hematophagous habits and the inherent complexity of its control in urban centers [1].

Several studies have drawn attention to natural products with larvicidal activity that could be useful in controlling many vectors [5-7], including *A. aegypti*[6,8,9]. The mode of action of natural insecticides is variable. While some of them inhibit normal growth and development [10-12], others inhibit the synthesis of tyrosinase, an enzyme involved in sclerotization of the cuticle, such as in the case of *Culex quinquefasciatus* larvae [13], or act as an antidiuretic hormone [14] and reduce reproductive capacity [15]. Plant extracts and phytochemicals have potential as products for mosquito control because many of them are selective, may often biodegrade to nontoxic products, and may be applied to mosquito breeding places in the same way as conventional insecticides [16-18]. Several studies have been carried out on lignans and their effects on insects [7,11,12,14,15,19], indicating that they could be larvicidal against *A. aegypti*[9]. The neolignans eupomatenoid-6, eupomatenoid-5 and conocarpan, isolated from the leaves of *Piper decurrens* A.DC. 1866 (Piperaceae), have demonstrated significant larval toxicity against *A. atropalpus* Coquillett, 1902 [20]. A potent reducing agent and antioxidant known as nor-dihydroguaiareticacid (NDGA), when added to the axenic larval medium or diet of adult mosquitoes of *A. aegypti,* has shown an increased life time (longevity) of adult insects [21].

Thus, the aim of this study was to determine the toxicity of burchellin and its morphological effects on the digestive system in immature forms (L3) of *A. aegypti.*

## Methods

### Isolation of burchellin

**Figure 1:**
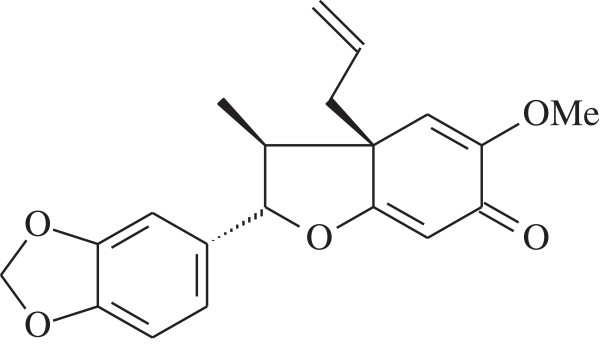
**Burchellin, neolignan from *Ocotea cymbarum *** **Kunth, Lauraceae.**

Burchellin derived from *O. cymbarum* collected in Belem, Para, was studied for its larvicidal activity. The botanical material was identified at Hamburg University, Germany, by Prof. Klaus Kubitzhi, and the neolignan burchellin (Figure 1) was purified from aerial parts of *O. cymbarum*[22]. A specimen from a small island of the lower Rio Negro, Amazonas, was collected and identified by Prof. K. Kubitzki, Hamburg. A voucher specimen (58576) was deposited at the Herbarium INPA, Manaus. Burchellin was isolated for the first time in 1972 from *Aniba burchelli*[23]. Its synthesis is described in the literature [24] and the compound was identified by ^1^H and ^13^C nuclear magnetic resonance. One-dimensional (^1^H and ^13^C) and two-dimensional (gHMQC, gHMBC, gCOSY and gNOESY) NMR analyses were performed on a Varian System spectrometer operating at 500 MHz (^1^H) and 125 MHz (^13^C). CDCl_3_ was used as the solvent with TMS (tetramethylsilane) as an internal standard. HRESIMS (high resolution electro spray ionization mass spectrometry) was carried out using a micrOTOF-II system from Bruker.

In this study, the dry and powdered stem (253 g) was extracted with hexane, dichloromethane and ethanol in a Soxhlet apparatus. The resultant solutions were filtered and concentrated in a rotary evaporator under reduced pressure, providing 4.2 g hexane extract, 1.90 g dichloromethane extract and 11.0 g ethanolic extract. The hexane extract was chromatographed on an H-60 silica gel column (Merck) using mixtures of chloroform and methanol of increasing polarity under pressure of 2 kgf/cm^2^ in a nitrogen atmosphere. Fractions 3 - 5 (411.9 mg) were pooled and rechromatographed on a silica gel 60 column (Merck) using a mixture of hexane and ethyl acetate of increasing polarity. Elution with hexane: ethylacetate (7:3) gave a fraction that was later subjected to HPLC, utilizing ethyl acetate: hexane (28:72) as the eluent, with a flow rate of 13 mL/min, and a Perkin-Elmer Si-6C column (250 × 16 mm, particle size of 10 μm), and UV detector at 260 nm [22]. The burchellin obtained was recrystallized from a mixture of hexane: ethylacetate and then with acetone, resulting in 287.1 mg of white crystals with a melting point of 150°C, as determined according to Araújo-Lima [23].

### Biosssays

*A. aegypti* eggs were obtained from the Vector Research and Support Center/NApVE (partnership DIRAC-IOC-VPAAPS), Oswaldo Cruz Institute, FIOCRUZ, Rio de Janeiro and were kept in the Diptera Laboratory of Oswaldo Cruz Institute, FIOCRUZ, Rio de Janeiro, in which a colony was also maintained. The bioassays were carried out using eggs that were placed in a receptacle containing mineral water with fish food (0.3 mg/larva) (Alcon Guppy) for hatching [9,25]. All experiments were carried out on third instar (L3) larvae (F1-F5) in triplicate with three repetitions, to determine the effect of the neolignan.

Burchellin was dissolved in acetone and diluted 1:4 in 0.15M NaCl at final concentrations of 0.001 – 300 ppm. The burchellin solutions were used in the treatment of the larval groups and individual larvae. In larval group treatment, with 10 larvae per group, burchellin was added to glass containers (4.0 cm × 4.5 cm) containing mineral water (10 mL) at final concentrations of 0.5, 1, 5, 10, 20, 30, 100, 200 and 300 ppm. In individualized larval treatment, with 20 individual larvae per group, the compound was added to glass containers (2.0 cm × 4.0 cm) containing mineral water (5 mL) at final concentrations of 0.001, 0.01, 0.1, 0.3, 0.5, 1, 3.8, 5, 10, 30, 50, 100, 150 and 300 ppm. *A. aegypti* larval (L3) groups and individuals (F1-F5) were evaluated in triplicate with three repetitions, as described elsewhere [9,25] and adapted from WHO [26]. Two control groups included one with acetone solution (without burchellin) and another with untreated solution. The bioassays were maintained in a climate-controlled chamber at 28 ± 1°C, 70 ± 10% relative humidity and 12-h photoperiod throughout the experiments, and toxicity against *A. aegypti* larvae and their growth development were evaluated until completely adults.

The data were analyzed using the ANOVA F-test [27] and χ^2^ test, where *P* ≤ 0.05 and *P* ≤ 0.01 were considered significant, respectively. Standard deviations were calculated using the averages from the experiments using GraphPad Instat 3.05 [28] and Trimmed Spearman-Karber analysis to determine the LC50 [29].

### Chemical stability in water vs biological activity

To evaluate toxicity according to the duration of the substance dissolved in medium used for rearing larvae, 15 ppm burchellin was added to mineral water without the presence of *A. aegypti* larvae*.* Ten larvae (L3) per group were added to the previously treated medium, according to the periods of incubation of the product (12, 24, 48, 72, 96 and 120 h), including the control groups. After 1 h exposure, the larvae were given fish meal (0.3 mg/mL). In case of mortality, the larvae were removed from the test medium.

To evaluate the duration of biological activity of the test compound in solution, burchellin at 30 ppm was added to the larval rearing medium in the presence of *A. aegypti* larvae (n = 100). After 1hexposure to the substance, the larvae were given fish meal (0.3 mg/mL). The larvae (n = 10), live or dead, were removed after different periods (1, 2, 3, 24, 48 and 76 h) of contact with the compound, and transferred to medium without burchellin, including control and acetone control. Larval mortality was assessed according to the time of exposure to the neolignan, to determine the minimal and maximal period of action of the compound on the *A. aegypti* larvae. All bioassays were carried out in a climate-controlled chamber at 28 ± 1°C, 70 ± 10% relative humidity and 12 h photoperiod.

### Histology - digestive system

A histological evaluation of the digestive system was performed using L3 larvae (treated, control and acetone control) fixed in 2.5% glutaraldehyde in sodium cacodylate buffer (0.1 M, pH 7.4) for 4 h. They were then dehydrated with increasing concentrations of ethanol (70, 80, 90, 96 and 100%), by immersion in each of these solutions for 15 min. Next, they were embedded in Historesin JB4 and the resultant blocks were sliced using a microtome to obtain a series of 3-μm thick sections. These sections were stained with hematoxylin-eosin, and then examined and photographed using a light microscope [30].

### Nitric oxide synthase (NOS) assay

Nitric oxide synthase activity was measured utilizing the protocol previously described [31]: total intestinal cells of the larvae (L3) of *A. aegypti* were recovered with trypsin/EDTA (0.05/0.02% v/v; Sigma Chemical Co.), washed and resuspended in 2 mL of HEPES buffer (pH 7.2; Sigma Chemical Co.). Protein concentration was 10.7 mg/mL as measured by spectrometry (260-280 nm). Next, the sample was mixed with a protease inhibitor cocktail, consisting of 0.1 mM phenylmethylsulfonyl fluoride (PMSF), 0.01% leupeptin, 0.2 mg/mL trypsin inhibitor and 1.0 mM benzamidine), in a final volume of 5 mL [31]. Aliquots of lysed cells were frozen in liquid nitrogen for later assay of NOS activity.

### NOS activity

In each assay, intestinal homogenate containing 200 μg/mL protein was mixed with the following reagents (all from Sigma Chemical Co.) in a 400-μL final volume: 0.2 mM NADPH, 360 μM L-arginine, 2 μM tetrahydrobiopterin, 1.0 μM FAD, 1.0 μM FMN, 0.3 mM CaCl_2_, 0.2 mM dithiothreitol and 50 mM potassium phosphate buffer (pH 7.4). In some samples, the constitutive NOS inhibitor L^ω^-nitro-L-arginine methyl ester (Sigma Chemical Co.), inducible NOS inhibitor diphenyliodonium chloride (Sigma Chemical Co.) and burchellin (1) (20 μg/mL) were added. A solution of acetone was used as a negative control and L-NAME as a positive control. NOS activity was determined in the reaction mixture by measuring the decrease in absorbance at 340 nm for 20 min continuously, and expressed as the amount of NADPH consumed during the enzymatic conversion of L-arginine to L-citrulline. Three independent experiments were performed, and the data obtained using different treatments were analyzed statistically by means of the Mann-Whitney test (P < 0.05).

## Results

Treatment of L3 larvae (in group) of *A. aegypti* with burchellin in rearing medium changed the period (in days) of larval and pupal development and emergence of adults at all concentrations tested when compared to the control groups and acetone control, mainly in the pupa phase of *A. aegypti*. Burchellin treatment in the group of L3 larvae caused a decrease in larval viability (L3 – L4) of 67% (6.7 ± 1.5) (P < 0.001), 63% (6.3 ± 1.1) (P < 0.001), 66% (6.6 ± 0.6) (P < 0.001) with concentrations of 5, 10 and 20 ppm, respectively. At 30 ppm, only 13% (1.3 ± 0.6) (P < 0.001) of L3 larvae were viable. The emergence of adults (L3 – adult) was 67% (6.7 ± 1.5) (P < 0.001), 60% (6.0 ± 1.0) (P < 0.001) and 40% (4.0 ± 1.0) (P < 0.001) at concentrations of 5- 20 ppm (Table 1A). The larvicidal activity of burchellin against *A. aegypti* was observed at a concentration of 30 ppm with 100%larval mortality between 24 and 78 h after application of the compound (Table 1B). The neolignan showed a larval mortality of 33 – 53% at concentrations of 5 -20 ppm (Table 1B). These data resulted in an LD_50_ of 15.5 ppm and LD_90_ of 27 ppm, using the trimmed Spearman-Karber method.

**Table 1:** **Viability (A) and mortality (B) of the *A. aegypti *** **group treated with burchellin**

**A**	**L3-L4**		**L4 - Pupae**		**Pupae -adult**		**L3-Adult**	
	**X ± SD**	**%**	**X ± SD**	**%**	**X ± SD**	**%**	**X ± SD**	**%**
Control	10.0 ± 0	100	10.0 ± 0 a	100	10.0 ± 0 a	100	10.0 ± 0 a	100
Control 2	10.0 ± 0	100	10.0 ± 0 ab	100	10.0 ± 0 ab	100	10.0 ± 0 ab	100
0.5 μg/mL	10.0 ± 0	100	10.0 ± 0 ab	100	10.0 ± 0 ab	100	10.0 ± 0 ab	100
1 μg/mL	10.0 ± 0	100	10.0 ± 0 ab	100	10.0 ± 0 ab	100	10.0 ± 0 ab	100
5 μg/mL	10,0 ± 0	67	6.7 ± 1.5 c***	100	6.7 ± 1.5 c***	100	6.7 ± 1.5 c***	67
10 μg/mL	10.0 ± 0	63	6.3 ± 1.1 c***	100	6.3 ± 1.1 cd***	95	6.0 ± 1.0 cd***	60
20 μg/mL	10.0 ± 0	66	6.6 ± 0.6 c***	71	4.7 ± 0.6 d***	85	4.0 ± 1.0 d***	40
30 μg/mL	10.0 ± 0	13	1.3 ± 0.6 d***	0	0	0	0	0
**B**	**Larvae**				**Pupae**			
	**X ± SD**		**VI**	%	**X ± SD**	**VI**	**%**	
Control	0		0	0	0	0	0	
Control 2	0		0	0	0	0	0	
0.5 μg/mL	0		0	0	0	0	0	
1 μg/mL	0		0	0	0	0	0	
5 μg/mL	3.3 ± 1.5 c**		3-3	33	0	0	0	
10 μg/mL	3.6 ± 1.2 c***		2-2	37	0.3 ± 0.6	7-7	5	
20 μg/mL	5.7 ± 0.2 c***		1-3	53	0.7 ± 0.6	7-7	14	
30 μg/mL	10 ± 0 d***		1-3	100	-	-	-	

Burchellin treatment on *A. aegypti* at 0.5 – 30 μg/mL concentration. Values are mean ± standard deviation (X ± SD), average of three replicates of 10 larvae L3 per each group. Values followed by the same letter did not significantly different from each other, P > 0.05 when the Tukey test was used. Significance levels are represented as ***P < 0.001 and **P < 0.01 vs. Control 2 = Acetone control.

The individual treatment of larvae (L3) with burchellin showed 100 to 30% larval viability (L3-L4), and emergence of adults at increasing concentrations of the test substance (0.001 and 0.5 ppm) (Table 2A). The same concentrations showed from zero to 70% larval mortality (Table 2B). These data indicated that the concentrations below 0.5 μg/mL did not have any larvicidal effect, but interfered with the development of the insect.

**Table 2:** **Viability (A) and mortality (B) of the *A. aegypti *** **individual treatment with burchellin**

**A**	**L3-L4**		**L4 - Pupae**		**Pupae -adult**		**L3-Adult**	
	**X ± SD**	**%**	**X ± SD**	**%**	**X ± SD**	**%**	**X ± SD**	**%**
Control	10.0 ± 0	100	10.0 ± 0a	100	10.0 ± 0a	100	10.0 ± 0a	100
Control 2	10.0 ± 0	100	10.0 ± 0ab	100	10.0 ± 0ab	100	10.0 ± 0ab	100
0.001 μg/mL	10.0 ± 0	100	10.0 ± 0ab	100	10.0 ± 0ab	100	10.0 ± 0ab	100
0.01 μg/mL	10.0 ± 0	100	10.0 ± 0ab	77	7.5 ± 0.6d***	100	7.5 ± 0.6d***	77
0.1 μg/mL	10.0 ± 0	100	10.0 ± 0ab	73	7.3 ± 0.6d***	100	7.3 ± 0.6d***	73
0.3 μg/mL	10.0 ± 0	100	10.0 ± 0ab	67	6.6 ± 0.6d***	100	6.6 ± 0.6d***	67
0.5 μg/mL	10.0 ± 0	100	10.0 ± 0ab	30	3.0 ± 1.0 c***	100	3.0 ± 1.0c***	30
**B**	**Larvae**				**Pupae**			
	**X ± SD**	**VI**	**%**		**X ± SD**		**VI**	**%**
Control	0	0	0		0		0	0
Control 2	0	0	0		0		0	0
0.001 μg/mL	0	0	0		0		0	0
0.01 μg/mL	2.4 ± 0.6c**	2-2	23		0		0	0
0.1 μg/mL	2.6 ± 0.6c**	2-2	27		0		0	0
0.3 μg/mL	3.3 ± 0.6c***	2-3	33		0		0	0
0.5 μg/mL	7.0 ± 1.0d***	2-3	70		0		0	0

Burchellin treatment on *A. aegypti* at 0.001 – 0.5 μg/mL concentration. Values are mean ± standard deviation (X ± SD), average of three replicates of 10 larvae L3 per each group. Values followed by the same letter did not significantly different from each other, P > 0.05 when the Tukey test was used. Significance levels are represented as ***P < 0.001 and *P < 0.05 vs. Control 2 = Acetone control. NO = not observed.

The treatment of individual larvae (L3) with the lignan at concentrations of 1 -10 ppm significantly reduced the development of the mosquitoes (L3- adult), resulting in only 27% (2.3 ± 1.2) (P < 0.001), 15% (1.3 ± 1.5) (P < 0.001), 8% (0.7 ± 0.6) (P < 0.001), and 7% (0.7 ± 0.6) (P < 0.001) adults (Table 3A). Larval mortality of *A. aegypti* was observed by toxicity of 63 to 90% (P < 0.001) for 1 - 10 ppm burchellin (Table 3B). The bioassays utilizing a concentration of 30 ppm showed the larvicidal activity of burchellin with 100% larval mortality from 24 to 72 h after treatment (P < 0.001) (Table 3B). The control groups and control appeared normal and did not show mortality. The data resulted in an LC_50_ of 0.4 ppm and LC_90_ of 5 ppm, using the trimmed Spearman-Karber method.

**Table 3:** **Viability (A) and mortality (B) of the *A. aegypti *** **individual treatment with burchellin**

**A**	**L3-L4**		**L4 - Pupae**		**Pupae -adult**		**L3-Adult**	
	**X ± SD**	**%**	**X ± SD**	**%**	**X ± SD**	**%**	**X ± SD**	**%**
Control	10.0 ± 0	100	10.0 ± 0 a	100	10.0 ± 0a	100	10.0 ± 0a	100
Control 2	10.0 ± 0	100	10.0 ± 0 ab	100	10.0 ± 0ab	100	10.0 ± 0ab	100
0.5 μg/mL	10.0 ± 0	87	8.7 ± 0.6 ab	35	3.0 ± 1.0 c***	100	3.0 ± 1.0 c***	30
1 μg/mL	10.0 ± 0	87	8.7 ± 0.6 ab	42	3.7 ± 2.0 c***	64	2.3 ± 1.2 c***	27
3.8 μg/mL	10.0 ± 0	90	9.0 ± 1.7 ab	22	2.0 ± 2.0 c***	67	1.3 ± 1.5 c***	15
5 μg/mL	10.0 ± 0	87	8.3 ± 1.2 ab	12	1.0 ± 1.0 c***	67	0.7 ± 0.6 c***	8
10 μg/mL	10.0 ± 0	27	2.7 ± 1.2 c***	38	1.0 ± 1.0c***	67	0.7 ± 0.6 c***	7
30 μg/mL	10.0 ± 0	20	2.0 ± 0 c***	0	0	0	0	0
**B**	**Larvae**			**Pupae**				
	**X ± SD**	**VI**	**%**	**X ± SD**	**VI**		**%**	
Control	0	0	0	0	0		0	
Control 2	0	0	0	0	0		0	
0.5 μg/mL	7 ± 1 c***	3-38	70	0	0		0	
1 μg/mL	7 ± 0c***	2-31	63	0	0		0	
3.8 μg/mL	8 ± 2 cd***	2-36	80	0.7 ± 1.2	22-22c		33	
5 μg/mL	9 ± 0 cd***	2-39	90	0.3 ± 0.6	18-18c		33	
10 μg/mL	9 ± 1 cd***	2-30	90	0.3 ± 0.6	28-28c		33	
30 μg/mL	10 ± 0 cd***	2-4	100	0	0		0	

Burchellin individual treatment on *A. aegypti* at 0.5 - 30 μg/mL concentration. Values are mean ± standard deviation (X ± SD), average of three replicates of 10 larvae L3 per each group. Values followed by the same letter did not significantly different from each other, P > 0.05 when the Tukey test was used. Significance levels are represented as ***P < 0.001 vs. Control 2 = Acetone control.

### Chemical stability in water vs biological activity

The activity assays of burchellin added to larval rearing medium for different periods of exposure showed a mortality of 17% at 12 h and 10% at up to 24 h of exposure. Burchellin did not demonstrate toxicity after 48 h of incubation in water, suggesting a possible loss of toxicity towards *A. aegypti* after 48 h in aqueous solution (Table 4A).

**Table 4:** **Evaluation of biological activity of burchellin against *A. aegypti *** **(L3)**

**A**	**Mortality (L3 larvae)**	
	**X ± SD**	**%**
Control	a	0
Control 2	b	0
12 h	1.66 ± 1.15 ab**	17
24 h	1.0 ± 0	10
48 h	1.0 ± 0	3
72 h	0	0
96 h	0	0
120 h	0	0
**B**	**Mortality (L3 larvae)**	
	**Number**	**%**
Control	0	0
Control 2	0	0
1 h	0	0
2 h	0	0
3 h	0	0
24 h	0	0
48 h	60	100

After 12-120 h (4A) and 1-48 h (4B) incubation, larvae in mid-development were treated with burchellin. Treatment of *A. aegypti* with 15 μg/mL burchellin using 10 L3 larvae per group (4A) and 30 μg/mL burchellin using 100 larvae (L3) per group (4B). Larvae (n = 10) were removed at intervals of 1 - 48 h (4B). All experiments were performed in triplicate. Values are mean ± standard deviation (X ± SD). Values followed by the same letter did not differ significantly; Tukey test, P > 0.05. Significance levels are represented as **P < 0.01 vs. Control 2 = Acetone control.

The burchellin absorption assays (hours) in larvae for different periods showed a 100% mortality only after exposure to the “medium + product” solution and for at least 48 h. These data suggest the need for exposure of larvae to a minimum period of 48 h for absorption of the test compound (Table 4B).

### Histomorphology

**Figure 2:**
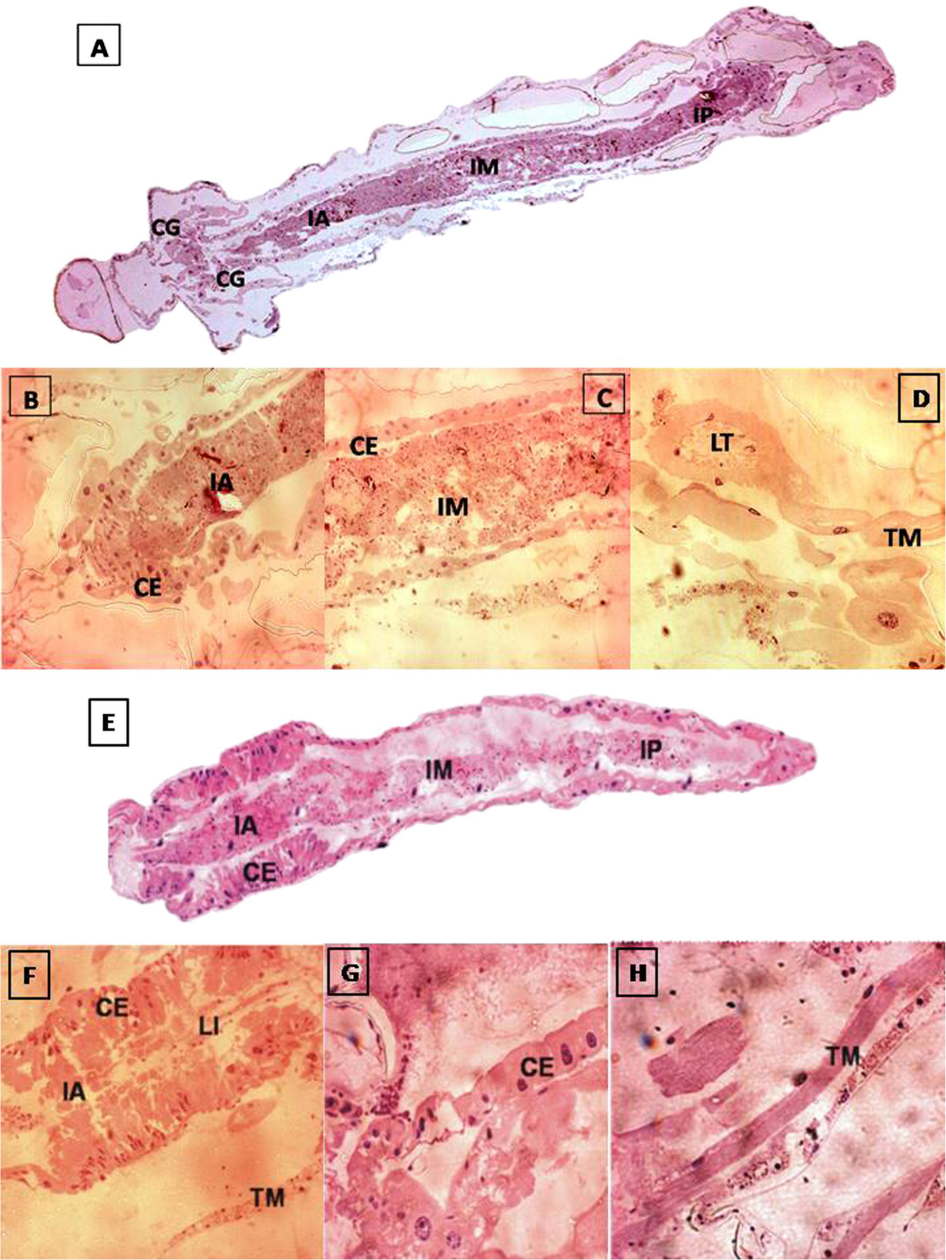
**Photomicrographs of the digestive tract of larvae of third instar of *A. aegypti *maintained with food.** Longitudinal sections stained with HE. A- D: control group. **A**. Overview. Gastric caeca (GC) foregut (IA), midgut (IM), hindgut (IP) 10×. **B**. Foregut details (IA) and intestinal epithelial cells (EC) 20×. **C**. Midgut (IM) and intestinal epithelial cells (EC) 20×. **C**. Detail of the Malpighian tubules (TM) and light of Malpighian tubule (LT) 20×. **E - H**: acetone group. **E**. Overview. Foregut (IA), midgut (IM), hindgut. (IP) and intestinal epithelial cells 20×. **F**. Foregut details (IA), intestinal lumen (LI) intestinal epithelial cells (EC) and muscle tissue (TM) 20×. **G**. Detail of intestinal epithelial cells (EC) 40×. H. Detail of muscle tissue (TM) 4×.

Histological analysis of the mosquito larvae of the control groups showed the three regions of the digestive tract (anterior, middle and posterior) with normal appearance and without morphological alterations. The epithelial cells of the anterior region were arranged in a single layer of short cylindrical cells and with an apical surface, demonstrating a thin line representing the brush border, also showing gastric caeca with normal appearance (Figure 2A, 2E). The peritrophic membrane showed a fine membrane completely surrounding all the alimentary content (Figure 2B, 2F). In the middle region of the intestine, the epithelial cells appeared a little taller with the cytoplasm preserved, while the brush border was a little thicker. This region contained some cells with a more globose appearance, similar to caliciform cells, and with a typical appearing nucleus (Figure 2C, 2G). The posterior region of the intestine showed for the most part cells with a laced appearance of the cytoplasm, displaying indentations and a thick brush border on the apical surface. Malpighian tubules and muscle tissue showed typical appearance (Figure 2D, 2H)).

**Figure 3:**
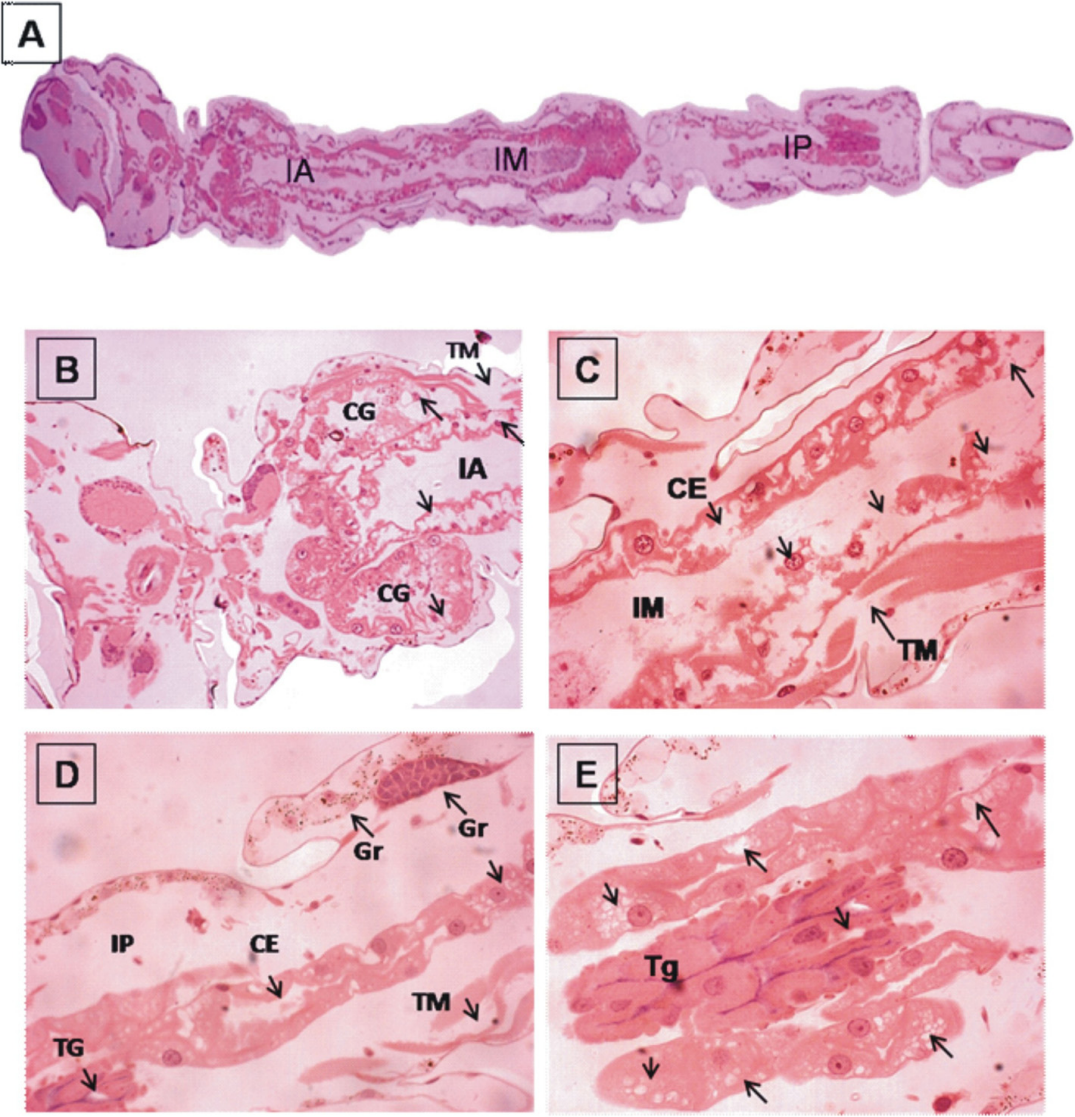
**Photomicrographs of the midgut of third instar larvae of *A. aegypti *maintained with food and treated with Burchellin.** Longitudinal sections stained with HE. **A**. Overview of foregut (IA), midgut (IM), hindgut (IP) 20×. **B**. Vacuolization in gastric caeca (CG) and cellular disorder, muscle tissue with breakpoints (TM) and vacuolated cells in the foregut showing spaces between them (IA) 40×. **C**. Midgut (IM), alteration of basal epithelial cells of the intestine (EC), break points of the muscle tissue (TM) 40×. **D**. Hindgut (IP), disorder of epithelial cells (EC), granule formation (arrows), disruption of muscle tissue (TM) and vacuoles in the Malpighian tubules (TG) 40×. **E**. Detail of the Malpighian tubules with vacuoles and intracellular spacing (TG) 40×.

The larvae treated with burchellin exhibited alterations in the middle intestine (Figure 3A, B, C) with evidence of cell destruction, vacuolization of epithelial cells, tissue disorganization with spacing between the cells and some rupture points of muscle tissue. There was an apparent accumulation of granules in some areas of the cytoplasm, and faint and/or absent nuclei (Figure 3D). Some cells showed a lack of cytoplasmic borders (Figure 3B, C, D and E). Alterations in gastric caeca were also observed with the presence of vacuoles and cellular disorganization. The Malpighian tubules of the larvae treated with burchellin also showed alterations, namely the presence of vacuolated cells, spacing between the cells and clear or absent nucleus (Figure 3B and E).

## Discussion

The results obtained in this study with the neolignan burchellin, isolated from the stem of *O. cymbarum*in the hexane fraction, demonstrated effective larvicidal activity against *A. aegypti*, indicating that the family Lauraceae harbors substances potentially bioactive against insects. Larvicidal activity of the neolignan with 50% mortality occurred at a concentration of 15.5 ppm and with 100% mortality at 30 ppm, justifying the interest in this class of compounds or in the plant family in question. Studies indicate the presence of different biological activity in the genus *Ocotea* and that biomonitoring phytochemistry vs activity [32] can be confirmed by biological activities of insects [11,12], especially in the case of burchellin [14,19].This was again confirmed in this study where burchellin showed toxicity in group and individual treatment of L3 larvae of *A. aegypti.*

With regard to the class of lignoids, neolignans have been shown to be effective against L1 larvae of *A. aegypti,* as evidenced by the larvicidal activity (LC50 = 2.37 μg/mL) of grandisin, a neolignan isolated from the leaves of *Piper solmsianum* (Piperaceae) [9]. Similar activities have also been found for the neolignans eupomatenoid-6, eupomatenoid-5 and conocarpan against *A. atropalpus* Coq. [20]. The lignans epi-sesartemin and diayangambin isolated from *Phryma leptostachya* L. [33] and *Piper fimbriulatum* C. DC. have shown larvicidal activity against *A. aegypti*[34].

Analyses of the spectral data of four aliquots did not show structural differences in the molecule up to a period of 48 h when compared to data obtained with the original sample. The compound only underwent structural rearrangement when heated to 70°C in an oven [35]. These data showed that burchellin is a very stable compound in water and that it degrades only when subjected to high temperatures. Assays of activity *vs* exposure period demonstrated that *A. aegypti* larvae need constant contact with the neolignan for 100% mortality of immature forms (L3) up to 48 h.

The histomorphological alterations in larvae treated with burchellin, which possibly resulted in the death of the larvae (L3-L4) of *A. aegypti*, could be observed in the middle region of the intestine, with cellular destruction and disorganization, spacing between cells and vacuolization of epithelial cells. Similar results as ours were obtained in histological analyses of *Culex nigripalpus* larvae infected by *Bacillus thuringiensis medellin* (Cry11Bb) [36] and in intestinal cells of larvae of *Aedes albopictus* infected with *B. thuringiensis var. israelensis (Bti)*[37]. The authors reported as signs of infection, the presence of rounded cells, cytoplasm with granules, clear or absent nucleus and extensive cytoplasmic vacuolization of epithelial cells of the mesentery of these larvae.

*A. aegypti* larvae treated with burchellin showed a large quantity of granules in some areas of the cytoplasm resulting in cell lysis. According to Snodgrass [38], the digestive cells of the mesentery of insects can generally participate actively in the processes of secretion and absorption. Disintegration of these cells occurs through the accumulation of granular material in the apical portion with release of this material into the intestinal lumen of the insect.

The mosquito larvae treated with the test substance also showed alterations in the gastric caeca, namely vacuolated cells, cellular disorganization with spacing between the cell membrane, and the nucleus being clear or absent. These tissue changes are not restricted to the use of chemicals since they were also observed in the gastric caeca and in the region of the Malpighian tubules of the larvae of *Culex nigripalpus* Theobald, 1901 exposed to baculovirus [36].

These histomorphological findings help us understand the toxicity of substances related to the site of action of burchellin in *A. aegypti* larvae; the resultant tissue degradation hampers the survival of the larvae*.*

In this study, the neolignan burchellin showed little degradation when added to water, making it even more attractive for its marketing as a natural alternative for the control of mosquitoes. On the basis of histomorphological analysis, burchellin demonstrated targeted interference, acting on small specific regions of the larva, helping us understand the toxicity of the substance related to the site of action of the neolignan. Thus, burchellin appears to be a potent biolarvicide of natural origin and a safe and stable compound, in the control of *A. aegypti*, the principal mosquito transmitter of dengue and urban yellow fever.

## Conclusion

The neolignan burchellin proved to be a strong candidate for a natural, safe and stable phytolarvicidal to be used in population control of *A. aegypti*.

## Competing interests

The authors declare that they have no competing interests.

## Authors’ contributions

MM and JOAN conceived the idea for the study and wrote the manuscript; MM, JOAN, ROAS and JRSM performed the experimental assays. JMBF and MCOC isolated burchellin; AEG provided critical comments and participated in protocol drafting, results analysis and preparation of the discussion. All authors approved the final version of this manuscript.
